# Integration of coal gasification and waste heat recovery from high temperature steel slags: an emerging strategy to emission reduction

**DOI:** 10.1038/srep16591

**Published:** 2015-11-12

**Authors:** Yongqi Sun, Seetharaman Sridhar, Lili Liu, Xidong Wang, Zuotai Zhang

**Affiliations:** 1 Department of Energy and Resources Engineering, College of Engineering, Peking University, Beijing 100871, P.R. China; 2 WMG, International Digital Laboratory, University of Warwick, Coventry CV4 7AL, UK; 3 Beijing Key Laboratory for Solid Waste Utilization and Management, College of Engineering, Peking University, Beijing 100871, P.R. China

## Abstract

With the continuous urbanization and industrialization in the world, energy saving and greenhouse gas (GHG) emission reduction have been serious issues to be addressed, for which heat recovery from traditional energy-intensive industries makes up a significant strategy. Here we report a novel approach to extract the waste heat and iron from high temperature steel slags (1450–1650 ^o^C) produced in the steel industry, i.e., integration of coal gasification and steel slag treatment. Both the thermodynamics and kinetics of the pertinent reactions were identified. It was clarified that the kinetic mechanism for gasification varied from A2 model to A4 model (Avrami-Erofeev) in the presence of slags. Most importantly, the steel slags acted not only as good heat carriers but also as effective catalysts where the apparent activation energy for char gasification got remarkably reduced from 95.7 kJ/mol to 12.1 kJ/mol (A2 model). Furthermore, the FeO in the slags was found to be oxidized into Fe_3_O_4_, with an extra energy release, which offered a potential for magnetic separation. Moreover, based on the present research results, an emerging concept, composed of multiple industrial sectors, was proposed, which could serve as an important route to deal with the severe environmental problems in modern society.

With the rapid developments in urbanization and industrialization in the world during the past few decades, the demand for a sustainable and reliable energy has experienced radically growth. Conventionally, recovering the waste heat from energy-intensive industrial sectors such as the steel industry is an important strategy to deal with this issue. Indeed, many advanced technologies^1^,^2^,^3^,^4^,^5^,^6^ have been introduced into the steel industry. However, there is considerable scope for further improvement with regard to the energy consumption and greenhouse gas (GHG) emission, especially in the context of global warming^7^,^8^. On the other hand, waste heat recovery from hot slags is believed to represent one of last substantial and undeveloped potentials of energy savings in the iron and steel sector^9^,^10^.

China has been the largest iron and steel producer since 1996 and the annual pig iron and crude steel outputs were 712 Mt and 823 Mt in 2014^11^, respectively. In detail, the crude steel productions in China and the world since 2004 are shown in Fig. 1a, and the emissions of GHG during steel manufacturing contribute significantly to the global warming. To illustrate this, during the steel making process in 2014, more than 120 Mt steel slags were discharged at 1450–1650 ^o^C, carrying substantial high grade thermal energy amounting to carbon equivalents of more than 7.1 Mt standard coal. However, the recovery ratio of the high value energy is less than 2% in China^12^ because of the fundamental constraints, namely low thermal conductivity, easy crystallization trend and discontinuous time-temperature availability^13^,^14^. This has necessitated the development of advanced approaches that can effectively meet these constraints. In this case, chemical methods have been proposed to provide promising routes because of the specific advantages including the combination of various industrial sectors and the production of high value syngas.

As for the heat recovery from blast furnace slags, several chemical methods have been proposed including biomass gasification^15^,^16^, coal gasification^17^,^18^,^19^ as well as methane deforming reaction^20^,^21^. As for the heat recovery from steel slags, developments of chemical methods on heat recovery from steel slags are quite limited^22^. The present study was therefore motivated and an integrated process was designed combining of coal gasification and heat recovery form steel slags. In 2013, the total production of raw coal in China was 3.56 Gt (gigatonnes)^23^, accounting for nearly half of the world’s production. The annual coal production is displayed in Fig. 1b in China and the world since 2003^24^, which indicate that the coal industry is faced with the serious situation with respect to the reduction of emissions. Compared with direct combustion, coal gasification has some individual advantages such as less pollution, high efficiency and deeper utilization of the product^25^. During the coal gasification process, the demanded heat is generally supplied by the partial combustion of the coal, from the viewpoint of which the steel slags, tapped at high temperatures, could act as alternative heat sources.

Furthermore, in the sense of carbon capture and storage (CCS), pure CO_2_ was employed as the reactive agent to conduct the gasification tests. The fundamental mechanism of coal gasification was identified taking into account the underlying thermodynamic and the kinetic factors. From the viewpoint of waste-to-clean energy strategy, it is expected that this emerging method could show substantial environmental and economic benefit in the near future. Additionally, it should be pointed out here that three samples were used in this study, i.e., a raw steel slag sample (**S0**), a raw coal sample (**S1**) and a mixture with the mass ratio of coal sample to steel slags of 1:1 (**S2**).

## Results

### Transient behavior of mass variation during coal gasification

**Figure 2:**
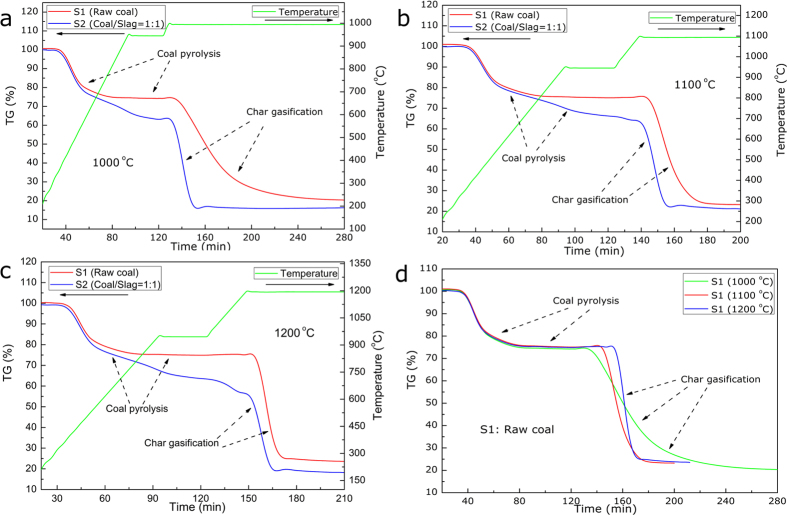
TG curves of the coal gasification process (a) TG curves at 1000 ^o^C, (b) TG curves at 1100 ^o^C, (c) TG curves at 1200 ^o^C and (d) TG curves of the raw coal (S1) at 1000, 1100 and 1200 ^o^C.

To characterize the transient behavior of the coal gasification, the mass evolutions of coal samples during the heating path were continuously measured using a thermo-gravimetric (TG) analyzer, as detailed in Fig. 2. As can be seen, the overall process of coal gasification could strictly be divided into two stages based on the TG curves, i.e., firstly, a coal pyrolysis stage for coal char preparation at <950 ^o^C in the atmosphere of N_2_ and, secondly, a char/CO_2_ gasification process at 1000–1200 ^o^C in the atmosphere of CO_2_. The coal gasification process, indeed, started with the low temperature pyrolysis of organic materials with a relatively low reaction rate. This step of coal pyrolysis started at around 300 ^o^C and got completed at around 900 ^o^C, during which the various covalent bonds were cleaved and the volatiles were decomposed; the gases including CO and H_2_ were thus released and the char was finally formed^26^,^27^. According to the ultimate analyses, the molecular formula of raw coal was deduced as CH_0.28_O_0.43_; thus this stage could be described by **equation** (1). Another phenomenon observed was that the steel slags influenced the stage of coal pyrolysis, as detailed in Fig. 2a–c. First, with the existence of slags a longer reaction time appeared, which could originate from an inhibitory effect of steel slags on the mass transfer steps in the samples. Second, with steel slags, more mass was consumed during the coal pyrolysis stage, which could indicate the occurrence of reactions between the char produced and the steel slags.


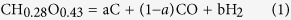


The most important stage was the gasification reaction between the char formed and CO_2_ agent at the set temperatures. Fig. 2d displays the gasification process of the raw coal and it can be seen that as the gasifying temperature increased from 1000 ^o^C to 1200 ^o^C, the reaction time required to complete the char gasification process was greatly shortened because of the enhanced reaction rate, i.e., from 130 min at 1000 ^o^C, 45 min at 1100 ^o^C to 25 min at 1200 ^o^C, respectively. Figure 2a–c displayed the mass variations of the raw coal and the coal/slags mixture during the coal gasification at temperatures of 1000 ^o^C, 1100 ^o^C and 1200 ^o^C, respectively. As can be observed, in contrast to the coal pyrolysis stage, the char/CO_2_ gasification process was remarkably enhanced by the steel slags as marked by a smaller reaction time and a larger slope of the TG curves, especially at 1000 ^o^C (Fig. 2a); whereas the gasification rates at 1100 ^o^C and 1200 ^o^C were also improved by the steel slags, albeit in relatively smaller increments (Fig. 2bc) because of the intrinsic high reaction rate at these temperatures.

**Figure 3:**
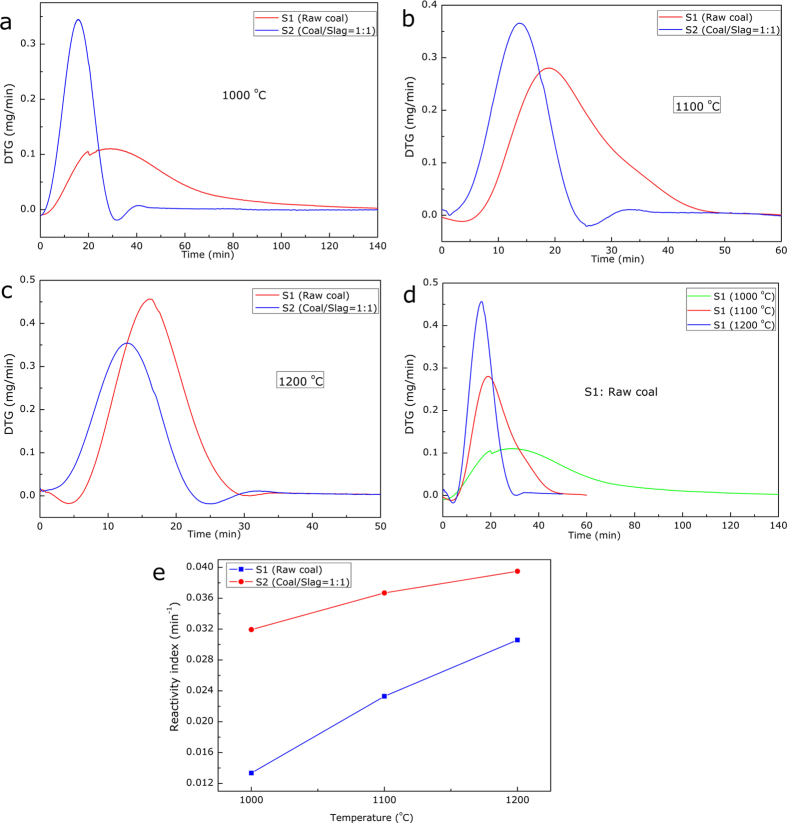
DTG curves of the coal gasification process (a) DTG curves at 1000 ^o^C, (b) DTG curves at 1100 ^o^C, (c) DTG curves at 1200 ^o^C, (d) DTG curves of S1 at 1000, 1100 and 1200 ^o^C and (e) reactivity indexes of the samples.

The differential thermal gravity (DTG) curves of the char/CO_2_ reaction stage could display the reaction rate of the gasification process more explicitly, as presented in Fig. 3a–d. As can be seen, for both the raw coal and the coal/slag mixture, the reaction rate was greatly enhanced with increasing gasifying temperature and furthermore, the presence of the steel slags resulted in a shorter reaction time. The latter, actually, could be indicative of a catalytic effect caused by the slags. Indeed, the variations of the reaction time could reasonably be described using a new constant, namely reactivity index, which could be defined as^28^,^29^:


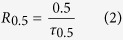


where *R*_*0.5*_ and *τ*_*0.5*_ are the reactivity index and time when reaction conversion ratio reaches 0.5. The variation trend of the reactivity index with varying samples and gasifying temperatures is presented in Fig. 3e, which clearly indicated that the increasing gasifying temperature and the existence of steel slags decreased the reaction time and consequently increased the reactivity index of the char/CO_2_ reactions.

### Kinetic mechanism of coal gasification

As the char/CO_2_ reaction was a typical reaction in the family of gas-solid reactions, various mechanism functions of gas-solid reactions could be employed to clarify the kinetic mechanism of this process^17^,^18^,^30^,^31^. The determination of the likely kinetic mechanisms should be based on two fundamental rules. First, the linear relationship between the integral function F(x) (where x represents the conversion degree of coal char) and *t*, i.e., the correlation coefficients (R^2^) of all plots, were compared and analyzed, as a larger correlation coefficient generally suggested a better result of model fitting. Second, the obtained mechanisms of the char/CO_2_ should be fully discussed and seriously compared with previously published results, because a deeper understanding of the mechanism was considered much more important than a simple linear ratio in terms of mathematics^32^.

**Figure 4:**
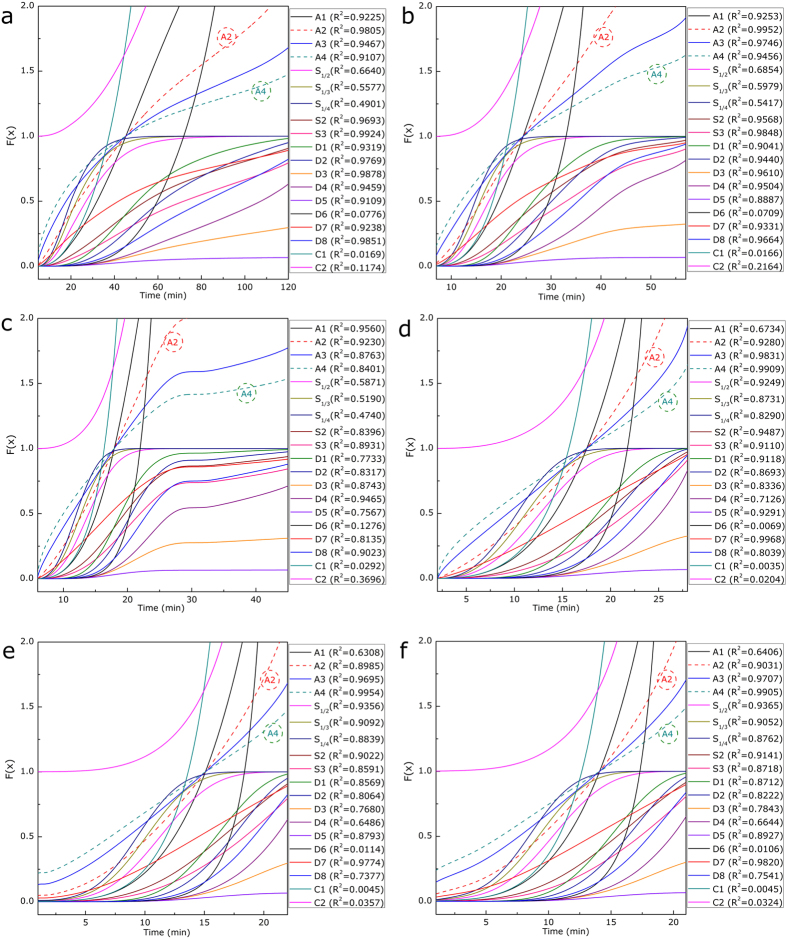
Kinetic models of char/CO_2_ reactions for different samples (a) S1 at 1000 ^o^C, (b) S1 at 1100 ^o^C, (c) S1 at 1200 ^o^C, (d) S2 at 1000 ^o^C, (e) S2 at 1100 ^o^C and (f) S2 at 1200 ^o^C.

The F(x) versus *t* plots were first established using the mechanism functions including nucleation growth, chemical reaction and mass diffusions (detailed in Supplementary Table S1), as presented in Fig. 4. The results demonstrated that, for sample **S1** without steel slags, an A2 model (Avrami-Erofeev, m = 2) could interpret the kinetic mechanism most reasonably, as described by **equation.** (3). In fact, Avrami-Erofeev models were generally used to interpret the gas-solid reactions when the porosity of the solids varies during the reactions, the rate-controlling step of which is the nucleation step^30^,^31^,^33^. The char/CO_2_ gasification process in this study could be scientifically described by an Avrami-Erofeev model because of the continuously varying porosity of the char as the reaction progressed, which was also in agreement with the previous studies^17^,^18^. As for mixture sample **S2**, it can be seen that the kinetic function was changed in the presence of steel slags, i.e., an A4 model could best characterize the kinetic mechanism, as described by **equation** (4), instead of an A2 model. To further prove the foregoing analysis, the plots results of the correlation coefficients (R^2^) and the apparent gasification rate constants (*k*) are detailed in Table 1 based on A2 and A4 model. It could clearly be concluded that the existence of the steel slags not only enhanced the reaction rate of the gasification process but also influenced the kinetic mechanisms.


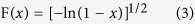



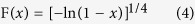


where 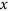 and 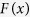 are the conversion degree of coal char and integral mechanism function, respectively.

As the kinetic mechanism changed from A2 model to A4 model because of the considerable effect of the steel slags, then the corresponding apparent rate constants (*k*) could be derived, as presented in Table 1. It should be pointed out that the kinetic models are clarified, not only from the viewpoint of mathematic relationship, but also from the viewpoint of basic mechanistic understanding and previous studies^17^,^18^,^31^,^32^,^33^. It is appreciated that the Avrami-Eroofeev model includes intrinsically gas phase transport along with chemical reaction kinetics but our approach is to extract, from the data apparent activation energy values and compare these to what might be physically expected for various rate limiting steps. According to the rate constants k, the apparent activation energy (*E*_*a*_) for char gasification could further be determined, as shown in Table 1. The activation energy for char gasification decreased prominently from 95.7 kJ/mol to 12.1 kJ/mol and 92.6 kJ/mol to 13.8 kJ/mol using the A2 model and A4 model, respectively, which indicated a remarkable catalytic effect of the steel slags on the char gasification. As the activation energy was decreased to 12.1 kJ/mol, the resistance for the Boudourad reaction was greatly reduced and thus the chemical-reaction rate was significantly enhanced. Under such a condition, it is likely that gas phase transport could account for the rate-controlling stage, compared with the stage of chemical reaction in the overall process. Indeed, gas phase diffusion would be expected to exhibit an activation energy of 4.2–21 kJ/mol^34^,^35^, which is lower than the corresponding values measured when the chemical reaction acts as the rate-controlling step with an activation energy generally more than 42 kJ/mol. As gas diffusion became a dominant step of the char gasification, if the amount of char sample used was increased, the flow rate of the reactive agent should be also adjusted accordingly, especially with the addition of slags.

### Syngas production and the thermodynamics

**Figure 5:**
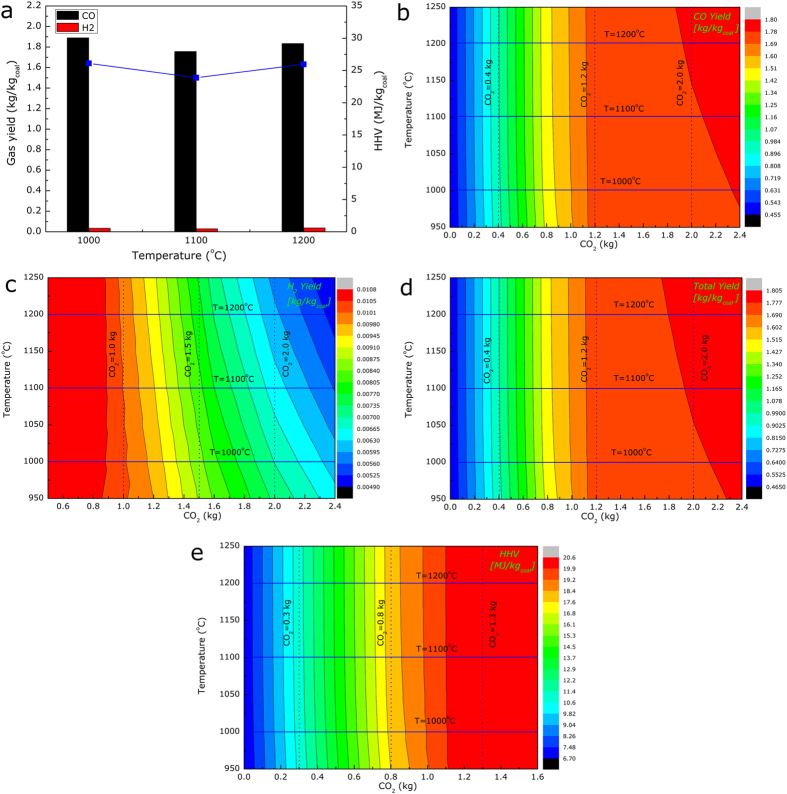
Thermodynamics and syngas production of coal gasification reactions (a) experimental gas yields, (b) CO yield by FactSage, (c) H_2_ yield by FactSage, (d) total gas yield by FactSage, and (e) HHV of the yielded gas by FactSage.

As one of the main objectives of the present study was to obtain the syngas composed of CO, H_2_ and other gases, the concentration of the syngas versus time was detected using a gas analyzer. As an example, the transient curve of the released CO gas for sample **S1** gasifying at 1000 ^o^C is presented in supplementary Figure S1a. It can be noted that the transient curve could be divided into two stages, i.e., a weak stage of coal pyrolysis and a strong stage of char gasification, which could exactly verify the overall trend of mass variation by TG measurements. It should be pointed out that no remarkable CH_4_ release was detected in the present experiments and therefore only the yields of CO and H_2_ were analyzed. Based on the transient concentration of CO and H_2_, the productions and the higher heating value (HHV) of the syngas could be deduced, as shown in Fig. 5a. It was found that the values of the yielded syngas almost kept constant, i.e., 1.60 Nm^3^ CO and 0.41 Nm^3^ H_2_ per kilogram of raw coal, respectively. The increasing temperature showed no influence on the final productions of CO and H_2_ but only improved the reaction rate because at the present temperatures (1000–1200 ^o^C), the gasification reaction could be completed and there was no residual organics in the samples.

To further clarify the thermodynamics of coal gasification and predict the syngas yields under various experimental conditions, the equilibrium syngas yields were calculated using FactSage software^36^. The calculated results including the contents of the syngas and its HHV are presented in Fig. 5b–e, based on which several characteristics could be clarified. First, the dominant components in the syngas were CO and H_2_, especially the CO gas. With increasing amount CO_2_ reacting during the gasification, the yield of CO remarkably increased as Boudourad reaction **(5)** would result in the production of two moles of CO gas for one mole of the reactant CO_2_ gas.


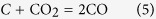


The yield of H_2_, on the other hand, showed an opposite trend and consequently, the total yield and HHV of the syngas increased significantly. However, the obtained syngas should be further separated after gasification as an increasing CO_2_ content in the syngas would be detrimental to the process. Thus the reactive CO_2_ amount should be reasonably controlled during an actual process. Second, the yields and the HHV of the syngas were almost constant with increasing temperature, which was in good agreement with the experimental results. Under a gasifying condition at high temperatures, the difference between various experiments lay mainly in the time required to complete the reaction but not the final equilibrium state; and therefore the equilibrium syngas yields did not change prominently. Third, the experimentally measured syngas yield almost equaled to the maximum values calculated by FactSage because under the present experimental conditions, the supplied gasifying agent of CO_2_ was excess.

## Discussion

**Figure 6:**
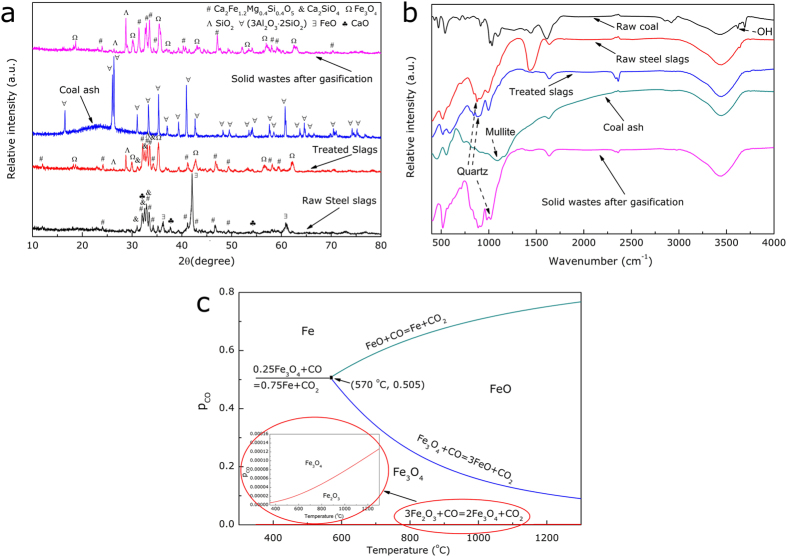
Characterizations of the char/CO_2_ reactions for different samples (a) XRD results of the mineral phases in the solids, (b) FTIR results of the mineral phases in the solids, and (c) Fe-CO-CO_2_ phase diagrams.

In order to further clarify the mechanism, the steel slags before and after heat-treatment (**S0**), the coal ash (**S1**) and the solid wastes after gasification (**S2**) were characterized using X-ray diffraction (XRD, D/Max 2500, Rigaku) and Fourier transformation infrared (FTIR) techniques, as presented in Fig. 6a,b. First, it can be noted that there was no residual char in the solid wastes, suggesting the complete reaction of the coal samples, which was also consistent with the thermodynamic results. Second, the coal ash was composed of glass phase and a mineral phase, namely mullite (3Al_2_O_3_·2SiO_2_), which could contribute to an important method for mullite preparation in the future. Third, in the steel slags, the main mineral phases were Ca_2_Fe_1.2_Mg_0.4_Si_0.4_O_5_, Ca_2_SiO_4_, CaO and FeO, which was, actually, in agreement with the catalytic effect of the slags. Firstly, CaO, as one of the mineral phases in the steel slags, was indeed a common catalyst for coal gasification to improve the rate the char/CO_2_ gasification^37^,^38^. Secondly, compared to Ca salts, the iron oxide in the slags could have a strong activity on the char gasification and a possible catalytic mechanism could be described by means of **equations (6–7)**^18^,^39^,^40^. On the other hand, Nishiyama^41^ reported that a low-rank coal is more sensitive to catalyst loadings than a high-rank coal due to the high concentrations of oxygen-containing functional groups, which was in agreement with the present results.


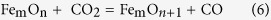



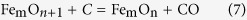


After the steel slags were treated, an interesting phenomenon was observed that more Fe_3_O_4_ crystalline phase appeared, either in the steel slags simply heat-treated in the CO_2_ atmosphere or in the solid wastes after gasification reactions at 1200 ^o^C. Fig. 6c presents the detailed phase diagram of Fe-CO-CO_2_ system, which could elaborate this phenomenon in theory. Under the present condition, the local experimental CO partial pressure and temperature were located in the Fe_3_O_4_ region. Therefore the FeO present was continuously oxidized by the CO_2_ according to **equation** (8) and the extra exothermic heat of this reaction, could be also recovered. In addition, this reaction could show some potential of CO_2_ footprint reduction. Moreover, a series of control experiments were performed where only the steel slags were heated in CO_2_ atmosphere and the formation of CO gas in the syngas was detected, as shown in supplementary Figure S1b, which experimentally proved the occurrence of **reaction (8)** and was compatible with thermodynamic calculations. The acquisition of the Fe_3_O_4_ enriched slags is of great significance for the further utilization of the steel slags since the Fe_3_O_4_ could be separated and recovered from the steel slags using magnetic separation method enabling a complete recycling of the steel slags.


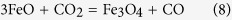


Based on the foregoing analysis, a roadmap for this novel method could be proposed, for which the technological process could be divided into several steps related to the individual industrial sectors. First, the high temperature steel slags were produced in the steel industry and broken into small granules to increase the surface area of the slags using various techniques such as an air granulation system or a roll type slag solidification system^42^. Second, the obtained granules were thoroughly contacted with the raw coal and the gasification reaction occurred in a reactor such as a fluidized bed in the atmosphere of CO_2_, during which the valuable syngas comprising CO and H_2_ was released. An alternative concept is that the thermal heat was first stored in high temperature phase change materials (PCMs)^43^ and then was utilized for coal gasification. Third, the syngas was desulfurized and the CO and H_2_ were separated from the syngas through different routes and utilized for various applications such as reactants in the DRI, raw materials in the chemical engineering industry and combustion for power generation. Fourth, the solid wastes should be timely and reasonably disposed and the Fe_3_O_4_ crystalline phase generated could be separated and recovered from the steel slags, which could be further used as raw materials in the steel industry. In brief, there were two specific advantages of this novel system, i.e., first not only the thermal heat but also the material resources of the steel slags were reasonably recovered and treated and second, several industrial sectors were combined and integrated in this big system.

**In summary**, in order to deal with the severe issues of increasing global warming and environmental degradation, we explored a novel route in this study, i.e., heat recovery from the high temperature steel slags through coal gasification reaction. The kinetic and thermodynamic aspects of the coal gasification were both determined. The results demonstrated that the steel slag showed multiple roles on the coal gasification phenomenon, i.e., not only an alternative heat carrier, but also an effective catalyst and even a reactant. Furthermore, it was found that the kinetic mechanism of coal char/CO_2_ gasification changed from A2 model to A4 model (Avrami-Erofeev) because of the presence of steel slags. Moreover, a big integrated system, composed of various industrial sectors, was proposed based on the identified results in this study.

## Methods

### Sample preparation

A low-rank coal sample was collected from Pingshuo power generation plant in Shanxi Province, China. Proximate analysis and ultimate analysis of the coal sample were performed using a thermogravimetric analyzer (TGA-701, LECO) and an elemental analyzer (vario Macro CHNS, Elementar), respectively, and the calorific value was determined by an adiabatic bomb calorimetry (Parr 6400 Calorimeter, Parr). The results obtained are summarized in Table 2. Additionally, the chemical compositions of the coal ash were determined by X-ray fluorescence (XRF) spectrometer (S4-Explorer, Bruker) and the results are detailed in Table 2. The coal sample was first dried for 24h at 105 ^o^C and then crushed and ground to a size smaller than 150 um.

Industrial steel slags were acquired from Shougang Corporation in Beijing, China, the chemical compositions of which were measured by XRF (S4-Explorer, Bruker) and displayed in Table 2. The slag was first crushed into small particles after drying, and then thoroughly mixed with the coal powder using a ball grinder for the subsequent gasification. Three samples were prepared using the foregoing materials, i.e., a raw steel slag sample (**S0**), a raw coal sample (**S1**) and a mixture with the mass ratio of coal sample to steel slags of 1:1 (**S2**), respectively.

### Apparatus and Procedure

A TG analyzer (S60/58341, Setaram) was adopted to perform the gasification tests, as depicted in supplementary Figure S2 in detail. The apparatus was mainly composed of two parts, i.e., a TG system and a syngas measurement system. During each gasification run, approximately 10 mg of sample was first placed in a Pt crucible with the height of 5 mm and diameter of 8 mm. Then, the coal sample was pyrolyzed for char preparation by heating from room temperature to 950 ^o^C at the rate of 10 ^o^C/min under the N_2_ flow (60 ml/min). After attaining the experimental temperature, the sample was held at constant temperature (950 ^o^C) for 30 min for thorough pyrolysis and after that, the char produced was heated to the set gasification temperature at the rate of 10 ^o^C/min. The experimental gasification temperatures were chosen as 1000 ^o^C, 1100 ^o^C and 1200 ^o^C. The samples were maintained at the experimental temperature for 5 min for attaining stabilization with respect to the temperature and gas atmosphere of the system. After this period, the N_2_ gas was replaced by the gasifying agent of CO_2_ with the flow rate 60 mL/min, which was enough to confirm the rapid proceeding of char/CO_2_ gasification. Then the char gasification occurred at the pre-set temperature, during which the variation of the sample mass was measured which enabled the identification of the kinetic mechanism of gasification. Further, to calculate and analyze the syngas yield, the composition of the syngas including concentrations of CO and H_2_ were measured by a gas analyzer (Testo pro350, Testo). In addition, to calibrate for the effects of buoyancy, blank runs were also performed.

### Methodology of kinetic analysis

The kinetic mechanism of coal gasification was characterized based on the TG analysis in this study. The rate of coal gasification could be described **equation** (9) and by rearranging and integrating **equation** (9) the integral mechanism function 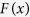 can be derived through **equation (10)**^30^,^31^.


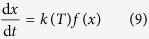



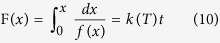


where 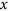, 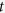, 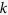, 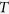, 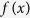 and 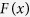 are the conversion degree of coal gasification, time, apparent gasification rate constant, absolute temperature, differential and integral mechanism function, respectively. Numerous mechanism functions have been developed to interpret gas-solid reactions, as provided in Supplementary Table S1. Moreover, these mechanism functions were employed and analyzed based on not only the mathematical optimum but also the proven mechanism and understanding related to coal gasification.

After the kinetic mechanism was determined, the apparent rate constants (*k*) for gasification could be deduced based on **equation** (10), and therefore the apparent activation energy of gasification *E*_*a*_ could be determined by means of Arrhenius equation, shown as **equation** (11).


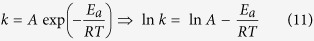


where *k*, *A*, *E*_*a*_, *R*, and *T* are the apparent gasification rate constant, pre-exponential factor, apparent activation energy of gasification, gas constant (8.314 J/mol/K), and absolute temperature (K), respectively. On these grounds the possible catalytic effect of the steel slags on the coal gasification reaction could be further clarified.

### Hypothesis of thermodynamics by FactSage

In this study, the effects of two variables, namely temperature and reactive agent (CO_2_), on syngas generation were explored at 1000–1200 ^o^C. In order to further analyze the syngas production and theoretically predict the syngas yield during gasification, equilibrium calculations were performed employing the approach of Gibbs free energy minimization using the FactSage software (FactSage 6.3)^34^. During calculations, the possible catalytic effect of the steel slags was not considered and the reactions were assumed to occur under isothermal (1100–1400 ^o^C) and isobaric (1 atm) conditions.

Furthermore, the coal compositions were simplified only including the elements of C, H and O. It was assumed that 1 kg coal reacted with the gasifying agent and therefore the amount of reacted organics was 0.7474 kg based on proximate analyses. The calculated equilibrium data were compared to the experimental data obtained by isothermal gasification and the thermodynamics of coal gasification was thus identified. In addition, the HHV of the syngas per mass of coal sample could be derived by means of **equation** (12) after the equilibrium syngas yields were obtained^33^,^44^. Additionally, it should be pointed out that the methodologies used in this study including the computations on kinetics and thermodynamics were general and could be expanded to other studies in the future such as gasification of carbonaceous feedstock.


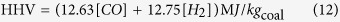


where [CO] and [H_2_] are the individual gas yields of CO and H_2_ per mass of raw coal.

## Additional Information

**How to cite this article**: Sun, Y. *et al.* Integration of coal gasification and waste heat recovery from high temperature steel slags: an emerging strategy to emission reduction. *Sci. Rep.* **5**, 16591; doi: 10.1038/srep16591 (2015).

## Supplementary Material

Supplementary Information

## Figures and Tables

**Figure 1:**
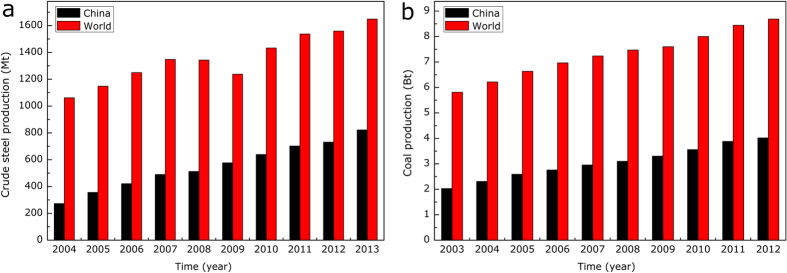
Annual crude steel and coal production in China and the world (a) crude steel production and (b) coal production.

**Table 1:** Kinetic mechanism of char/CO_2_ gasification and the derived kinetic parameters.

Kinetic Model	A2				A4			
	Avrami-Erofeev (m=2)				Avrami-Erofeev (m=4)			
Integral function	[−ln(1−x)]^1/2^				[−ln(1−x)]^1/4^			
Sample	*T*/^o^C	*k*/min^−1^	R^2^	*E*_*a*_/kJ mol^−1^	*T*/^o^C	*k*/min^−1^	R^2^	*E*_*a*_/kJ mol^−1^
**S1**	1000	0.01796	0.9805	95.7	1000	0.00895	0.9107	92.6
	1100	0.05381	0.9952		1100	0.02590	0.9456	
	1200	0.05999	0.9230		1200	0.02874	0.8401	
**S2**	1000	0.09571	0.9280	12.1	1000	0.05371	0.9909	13.8
	1100	0.10628	0.8985		1100	0.06145	0.9954	
	1200	0.11164	0.9031		1200	0.06397	0.9905	

**Table 2:** Characteristics of used materials in this study.

	Proximate analysis/%				Ultimate analysis/%					HHV (MJ/kg)
	Moisture	Volatile	Ash	Fixed carbon	C	H	O*	N	S	
Raw coal	3.79	31.61	21.47	45.93	60.58	1.41	35.08	1.29	1.64	24.21
Coal ash (XRF)	SiO_2_	Al_2_O_3_	Fe_2_O_3_	CaO	TiO_2_	MgO	K_2_O	Na_2_O		
	47.9	42.8	4.68	2.13	1.17	0.59	0.33	0.14		
Steel slags (XRF)	CaO	Fe_2_O_3_	SiO_2_	MgO	Al_2_O_3_	MnO	P_2_O_5_	TiO_2_	V_2_O_5_	Fe^2+^/TFe
	41.90	22.57	15.60	7.26	4.10	4.00	1.75	1.42	0.62	0.62

^*^Calculated by difference.
