# Supplementary material for: Integration of coal gasification and waste heat recovery from high temperature steel slags: an emerging strategy to emission reduction
Source: Sci Rep. 2015 Nov 12;5:16591. doi: 10.1038/srep16591 (PMC4642314; doi:10.1038/srep16591)

Integration of coal gasification and waste heat recovery from high temperature steel slags: an emerging strategy to emission reduction

Supplementary Information

Yongqi Sun[1], Seetharaman Sridhar [2], Lili Liu[1], Xidong Wang[1,3] and Zuotai Zhang[1,3*]

[1] Department of Energy and Resources Engineering, College of Engineering, Peking University, Beijing 100871, P.R. China, [2] WMG, International Digital Laboratory, University of Warwick, Coventry CV4 7AL, UK, [3] Beijing Key Laboratory for Solid Waste Utilization and Management, College of Engineering, Peking University, Beijing 100871, P.R. China

Supplementary Table S1. Various Gas-Solid kinetic mechanism functions, which could be divided into four types, i.e., the Avrami-Erofeev models, the Shrinking core models, the Diffusion models and the Chemical reaction models.

No	Kinetic mechanism	Differential function: f(x)	Integral function: F(x)
A_m	Avrami-Erofeev	$m(1-x)[-\ln(1-x)]^{m-1/m}$	$[-\ln(1-x)]^{1/m}$
A_1	m=1	1-x	$-\ln(1-x)$
A_2	m=2	$2(1-x)[-\ln(1-x)]^{1/2}$	$[-\ln(1-x)]^{1/2}$
A_3	m=3	$3(1-x)[-\ln(1-x)]^{2/3}$	$[-\ln(1-x)]^{1/3}$
A_4	m=4	$4(1-x)[-\ln(1-x)]^{3/4}$	$[-\ln(1-x)]^{1/3}$
S_m	Shrinking core	$m(1-x)^{m-1/m}$	$1-(1-x)^{1/m}$
$S_{1/2}$	m=1/2	$(1/2)(1-x)^{-1}$	$1-(1-x)^2$
$S_{1/3}$	m=1/3	$(1/3)(1-x)^{-2}$	$1-(1-x)^3$
$S_{1/4}$	m=1/4	$(1/4)(1-x)^{-3}$	$1-(1-x)^4$
S_2	m=2	$2(1-x)^{1/2}$	$1-(1-x)^{1/2}$
S_3	m=3	$3(1-x)^{2/3}$	$1-(1-x)^{1/3}$
D_m	Diffusion model		
D_1	one-dimensional	$1/2x^{-1}$	x^2
D_2	two-dimensional	$[-\ln(1-x)]^{-1}$	$x+(1-x)\ln(1-x)$
D_3	three-dimensional	$(3/2)(1-x)^{2/3}[1-(1-x)^{1/3}]^{-1}$	$[1-(1-x)^{1/3}]^2$
D_4	three-dimensional	$(3/2)[(1-x)^{-1/3}-1]^{-1}$	$1-2/3x-(1-x)^{2/3}$
D_5	3-D (anti-Jander)	$(3/2)(1+x)^{2/3}[(1+x)^{1/3}-1]^{-1}$	$[(1+x)^{1/3}-1]^2$
D_6	3-D (ZLT)	$(3/2)(1-x)^{4/3}[(1-x)^{-1/3}-1]^{-1}$	$[(1-x)^{-1/3}-1]^2$
D_7	3-D (Jander)	$6(1-x)^{2/3}[1-(1-x)^{1/3}]^{1/2}$	$[1-(1-x)^{1/3}]^{1/2}$
D_8	2-D (Jander)	$(1-x)^{1/2}[1-(1-x)^{1/2}]^2$	$[1-(1-x)^{1/2}]^2$
C_n	Chemical reaction	$(1-x)^n$	$(1-(1-x)^{1-n})/(1-n)$
C_2	n=2	$(1-x)^2$	$(1-x)^{-1}-1$
$C_{3/2}$	n=3/2	$2(1-x)^{(3/2)}$	$(1-x)^{-1/2}-1$

Supplementary Figure S1. Transient behaviors of CO release during coal gasification reactions: (a) Sample **S1** (raw coal) gasifying at 1000 ^{o}C and (b) Sample **S0** (raw steel slags) gasifying at 1000 ^{o}C.

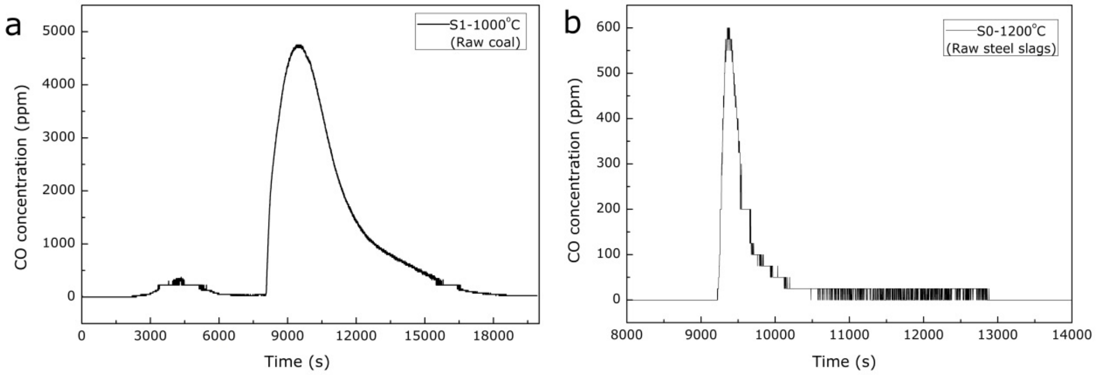

Supplementary Figure S2. Schematic of coal gasification system, which could be divided into two parts: a TG analyzers system and a gas cleaner and analyzer system.

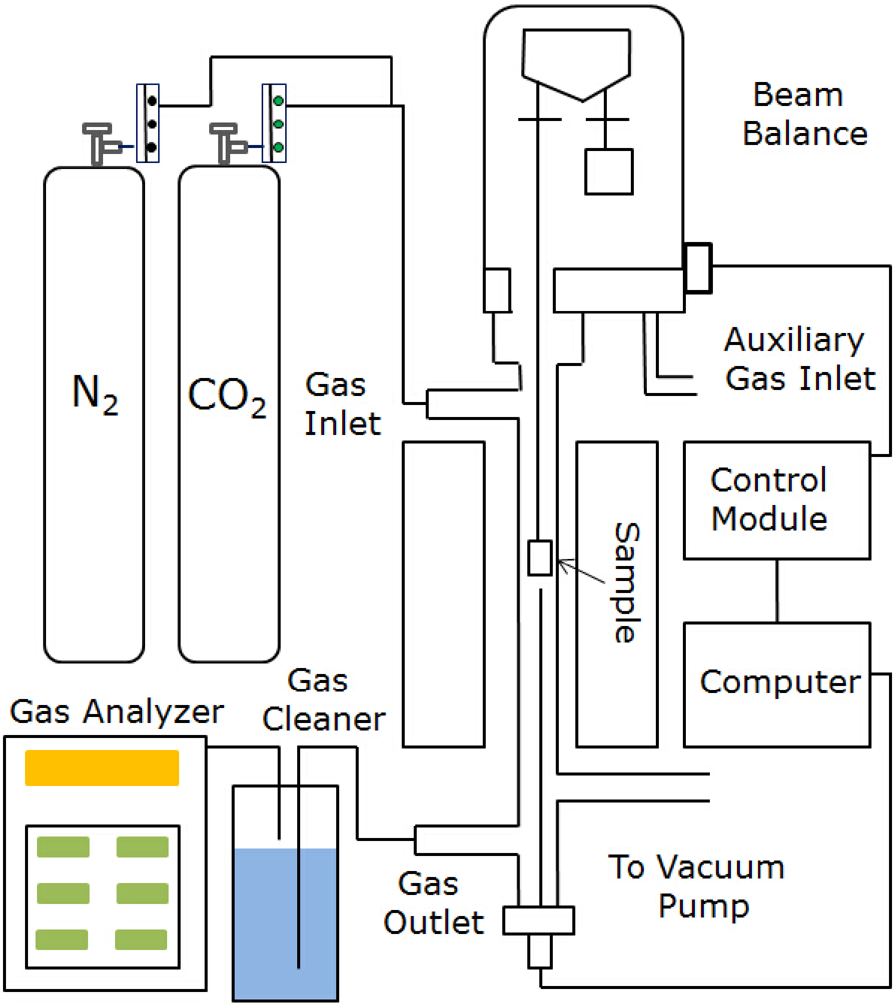

Supplement: Supplementary Information [file srep16591-s1.pdf]
